# Complete Genome Sequence of the *Anabaena* Myophage Elbi

**DOI:** 10.1128/MRA.00552-21

**Published:** 2021-07-22

**Authors:** Ria Patel, Julia Chen, Julie Xu, Emily Erdmann, Zoephia Laughlin, Richard M. Alvey

**Affiliations:** a Department of Biology, Illinois Wesleyan University, Bloomington, Illinois, USA; Montana State University

## Abstract

Here, we report the genome sequence of bacteriophage Elbi, which infects the cyanobacterium *Anabaena* sp. strain PCC 7120, a model organism for prokaryotic multicellular development. The 68,626 bp encode 108 proteins, of which 31 can be assigned a function. Elbi is similar to two *Anabaena* myophages, namely, A-1 and N-1, isolated in the 1970s.

## ANNOUNCEMENT

**FIG 1:**
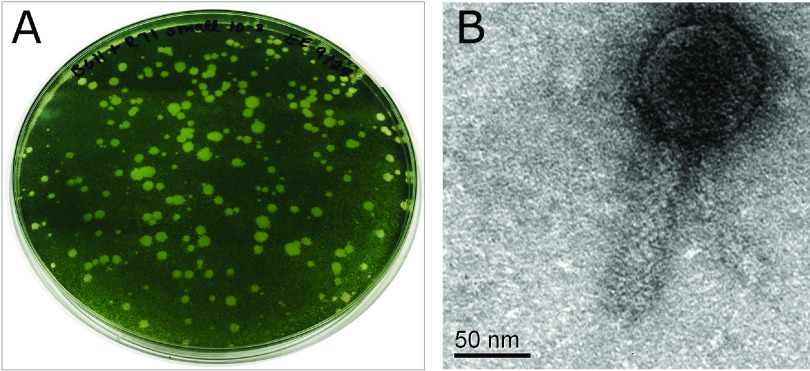
Elbi plaque and virion morphologies. (A) A lawn of *Anabaena* showing plaques formed by Elbi. (B) Transmission electron micrograph of Elbi revealing a *Myoviridae* morphology.

Despite their potential to manipulate freshwater cyanobacterial model systems, cyanobacteriophages have remained largely unexplored for these hosts. Here, we report the complete genome sequence of the cyanobacteriophage Elbi, which lyses vegetative cells of *Anabaena* sp. strain PCC 7120 (Fig. 1A). Elbi was isolated in July 2018 from the Des Plaines River near Oak Park, IL, through enrichment with a 3-day-old continuously lit culture at 30°C. After incubation of equal parts water sample, culture, and fresh media for an additional 4 days, a sample was filtered and plated with cells onto a BG-11 soft-agar overlay. Plaques appeared after 48 h of incubation. Transmission electron micrographs of phage particles mounted on carbon and Formvar grids negatively stained using 2% uranyl acetate revealed a *Myoviridae* morphology (Fig. 1B). Following several single-plaque isolations, DNA was extracted from the high-titer lysate using the Wizard DNA cleanup kit (Promega). A library was prepared using the Truseq Nano DNA library kit (Illumina), with Covaris shearing. Genome sequencing was performed using an Illumina MiSeq instrument at the North Carolina State University Genomic Sciences Laboratory. The resulting 150-bp single-end reads were assembled using Newbler v.2.9 and assessed for quality using Consed v.29 (1). This procedure resulted in three contigs whose connectivity was resolved using PCR and Sanger sequencing to yield a 68,626-bp contig with 3,352× coverage, a GC content of 36.7%, and circularly permuted, terminally redundant ends (2). Annotation was done using DNA Master v.5.23.6 (3) and Pecaan v.20210526 (https://blog.kbrinsgd.org/), with GeneMark v.3.25 (4), GLIMMER v.3.02 (5), NCBI BLAST v.2.9.0 (6), tRNAscan-SE v.2.0 (7), ARAGORN v.1.2.38 (8), CRISPRFinder v.2017-05-09 (9), HHpred v.3.2.0 (10), and Phamerator v.326 (11). Default parameters were used for all software.

Elbi gene content is most similar to that of two recently sequenced *Anabaena* myoviruses isolated in the 1970s, namely, A-1 and N-1 (12, 13). Both have similarly sized genomes and similar GC contents (Table 1). The DNA Master-calculated average nucleotide identities between Elbi and A-1 and N-1 are 71.3% and 70.1%, respectively. Likely functions were assigned to 31 of 108 identified genes. Phamerator revealed that 64 Elbi genes are shared with both phages, while 9 are shared with just A-1, 9 more are shared with just N-1, and 21 are unique. A-1 and N-1 have 32 and 19 unique genes, respectively. Although gene arrangement is identical for many of their shared genes, Elbi contains a large region of inversion (29,932 bp) flanked by 504-bp inverted repeat sequences. Repeats are found in the same relative locations in A-1 and N-1, although there does not appear to be substantial similarity between any of them. N-1 was previously reported to have a CRISPR array not present in A-1 and no similar array is in Elbi (12).

**TABLE 1:** Comparison of the Elbi genome with those of A-1, N-1, and *Anabaena* PCC 7120

Organism	Genome length (bp)	GC content (%)	No. of ORFs^*a*^	No. of CRISPR arrays
Elbi	68,626	36.7	108	0
A-1	68,304	36.5	112	0
N-1	64,690	35.4	98	1
*Anabaena* PCC 7120	7,211,789	41	6,132	9

a ORFs, open reading frames.

The only other publicly available genome for an *Anabaena* bacteriophage is for the *Podoviridae* A-4L, which was also isolated in the 1970s (14). Phamerator analysis suggests that A-4L does not share any closely related genes with the other three *Anabaena* phages.

### Data availability.

The complete genome sequence of phage Elbi is available in GenBank under the accession number MZ078141 with the NCBI SRA accession number SRX10797399.
